# Case Report: Pulmonary mixed infection by *Nocardia cyriacigeorgica*, *Stenotrophomonas maltophilia*, and *human cytomegalovirus* in a patient with minimal change nephrotic syndrome

**DOI:** 10.3389/fimmu.2025.1599958

**Published:** 2025-05-26

**Authors:** Yahua Li, Jiaqing Ye, Chenfeng Zhang, Weili Gao, Hong Zhang, Cuiying Zheng, Zhongjun Feng, Minghui Song, Jiahao Hao, Huifen Zuo, Zhenjun Zhao, Yumei Guo, Lijie Zhang

**Affiliations:** ^1^ Department of Respiratory and Critical Care Medicine, Hebei Medical University Third Hospital, Shijiazhuang, China; ^2^ Laboratory Department, Hebei Medical University Third Hospital, Shijiazhuang, China; ^3^ Hebei Key Laboratory of Intractable Pathogens, Shijiazhuang Center for Disease Control and Prevention, Shijiazhuan, China; ^4^ Laboratory Department, Hebei Yiling Hospital, Shijiazhuan, China

**Keywords:** metagenomic next-generation sequencing, pulmonary infection, *Nocardia cyriacigeorgica*, *Stenotrophomonas maltophilia*, *human cytomegalovirus*, minimal change nephrotic syndrome

## Abstract

To our knowledge, this is the first reported case of a pulmonary mixed infection involving *Nocardia cyriacigeorgica*, *Stenotrophomonas maltophilia*, and *human cytomegalovirus* (HCMV) in a patient with minimal change nephrotic syndrome (MCNS), which is of great clinical significance. We report the case of an 18-year-old male with a two-month history of MCNS who was admitted due to fever, cough, and bright red hemoptysis. Upon admission, he was treated with piperacillin/tazobactam and moxifloxacin for one week; however, the therapeutic response was suboptimal. Metagenomic Next-Generation Sequencing (mNGS) and microbiological culture of bronchoalveolar lavage fluid identified a pulmonary mixed infection involving *N. cyriacigeorgica*, *S. maltophilia*, and HCMV. Following the initiation of combination therapy with linezolid, trimethoprim-sulfamethoxazole, and ganciclovir, the patient’s condition improved markedly, and he was discharged in a stable condition. One-year follow-up revealed complete recovery with no recurrence. This case highlights the critical role of incorporating advanced molecular diagnostic tools such as mNGS into clinical practice and the need to be vigilance about opportunistic infections involving multiple pathogens, especially in patients receiving immunosuppressive therapy.

## Introduction

Minimal change nephrotic syndrome (MCNS) is one of the most common causes of idiopathic nephrotic syndrome (NS) (1). Treatment typically involves the use of corticosteroids and immunosuppressants, which can induce immunosuppression and elevate the risk of opportunistic infections caused by pathogenic microbes (2). *Nocardia cyriacigeorgica*, *Stenotrophomonas maltophilia*, and *human cytomegalovirus* (HCMV) are opportunistic pathogens, with an increased risk of infection in patients with impaired immune function or preexisting pulmonary diseases. Co-infections involving these three pathogens are exceedingly rare, with no documented cases reported in the literature. This report describes a case of a young adult with MCNS who developed a mixed pulmonary infection involving *N. cyriacigeorgica*, *S. maltophilia*, and HCMV, identified through metagenomic next-generation sequencing (mNGS) and bacterial culture of bronchoalveolar lavage fluid (BALF). Following targeted antimicrobial therapy, the patient achieved full recovery and was discharged.

## Case presentation

**Figure 1:**
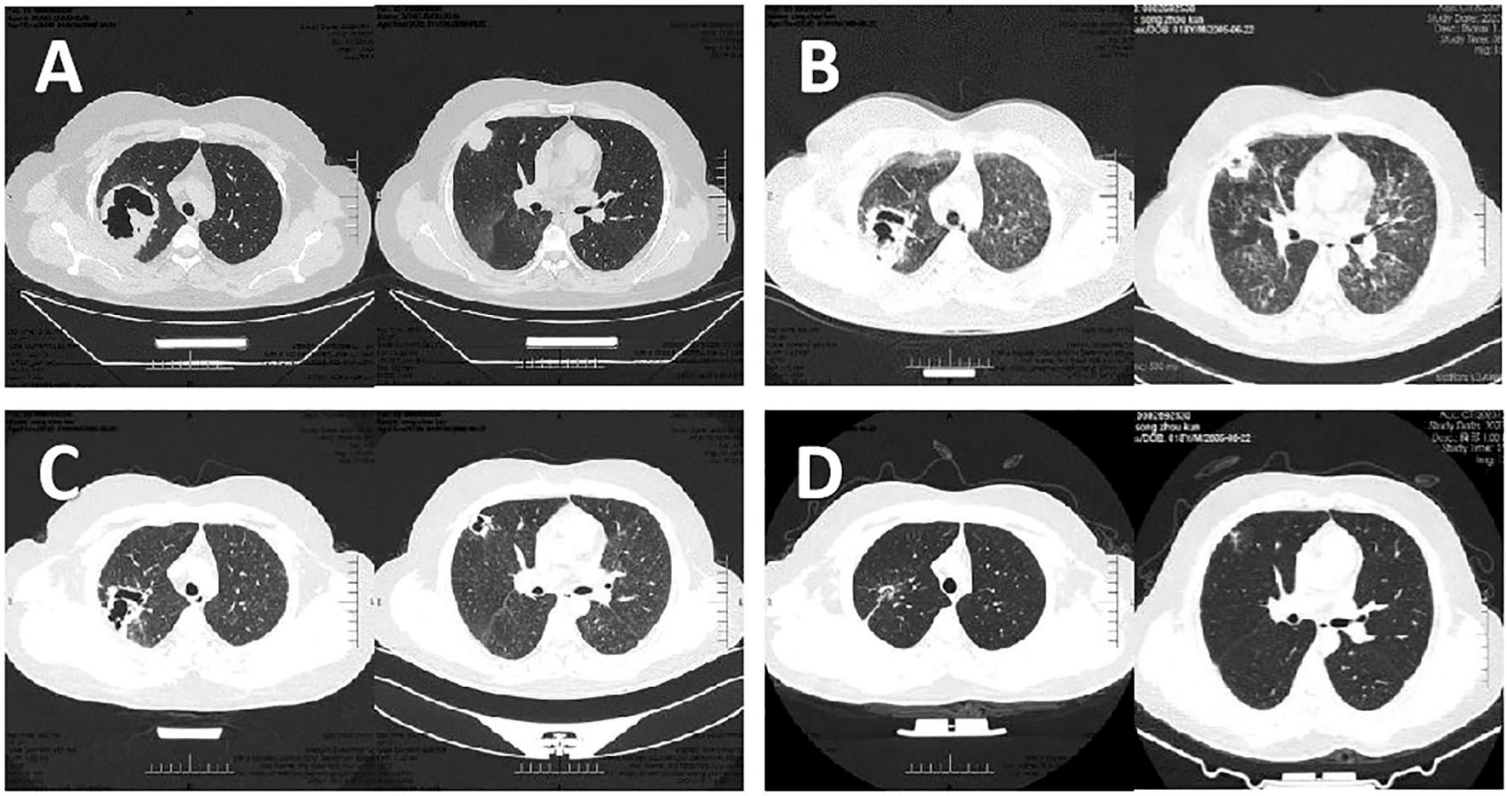
Results of Chest CT at different stages of the desease. **(A)** Chest CT showed a large patchy high-density lesion in the right upper lobe, associated with a large cavity. Scattered small patchy high-density lesions were also noted in the apical segment of the right upper lobe, some with small cavities. **(B)** Chest CT revealed the emergence of multiple diffuse, small patchy high-density lesions with poorly defined borders in both lungs, along with the development of multiple cavities. **(C)** Chest CT demonstrated a slight reduction in the size of the patchy high-density lesion in the right upper lobe apex compared to the prior examination. Diffuse ground-glass opacities in both lungs showed signs of absorption, with a reduction in cavity sizes. **(D)** Chest CT showed gradual shrinkage and closure of multiple nodules and cavities.

An 18-year-old male was admitted on September 11, 2023, presenting with fever, cough, and expectoration of bright red, blood-tinged sputum. Two months prior to admission, he had been diagnosed with MCNS and treated with methylprednisolone (40 mg once daily) and furosemide (20 mg twice daily). Upon admission, the prednisone dosage was reduced to 20 mg once daily due to severe infection and was discontinued two days later. Physical examination revealed a moon face, coarse breath sounds bilaterally, and absence of audible rales. Laboratory investigations revealed the following: 24-hour urinary protein excretion of 15.46 g/24 h, serum albumin of 20 g/L, total cholesterol of 8.85 mmol/L, triglycerides of 3.04 mmol/L, and a complete blood count showing a white blood cell (WBC) count of 15.47×10^9^/L with a neutrophil percentage of 86.20%. High-sensitivity C-reactive protein (Hs-CRP) was elevated to 116.81 mg/L, interleukin-6 (IL-6) to 33.3 pg/mL, blood glucose to 9.82 mmol/L, erythrocyte sedimentation rate to 73 mm/h, and sputum smear demonstrated Gram-negative rods. Renal histopathology findings were consistent with MCNS. Chest computed tomography (CT) revealed a large high-density area with cavitation in the right upper lobe, along with scattered small patchy opacities and multiple small cavities in the apical segments (**Figure 1A**). The patient’s maximum recorded body temperature was 39.1°C. Despite one week of empirical antimicrobial therapy with piperacillin/tazobactam and moxifloxacin, the patient’s condition persisted, characterized by fever, worsening cough, decreased hemoptysis, production of small amounts of white, viscous sputum, and the onset of dyspnea. Although WBC levels decreased to 9.89×10^9^/L, IL-6 levels increased significantly to 69.864 pg/mL, and arterial blood gas analysis indicated mild acute respiratory distress syndrome (ARDS) with an oxygenation index of 295 mmHg. Chest CT performed on September 18 revealed new diffuse multifocal patchy high-density areas and multiple cavity formations in both lungs (**Figure 1B**).

**Figure 2:**
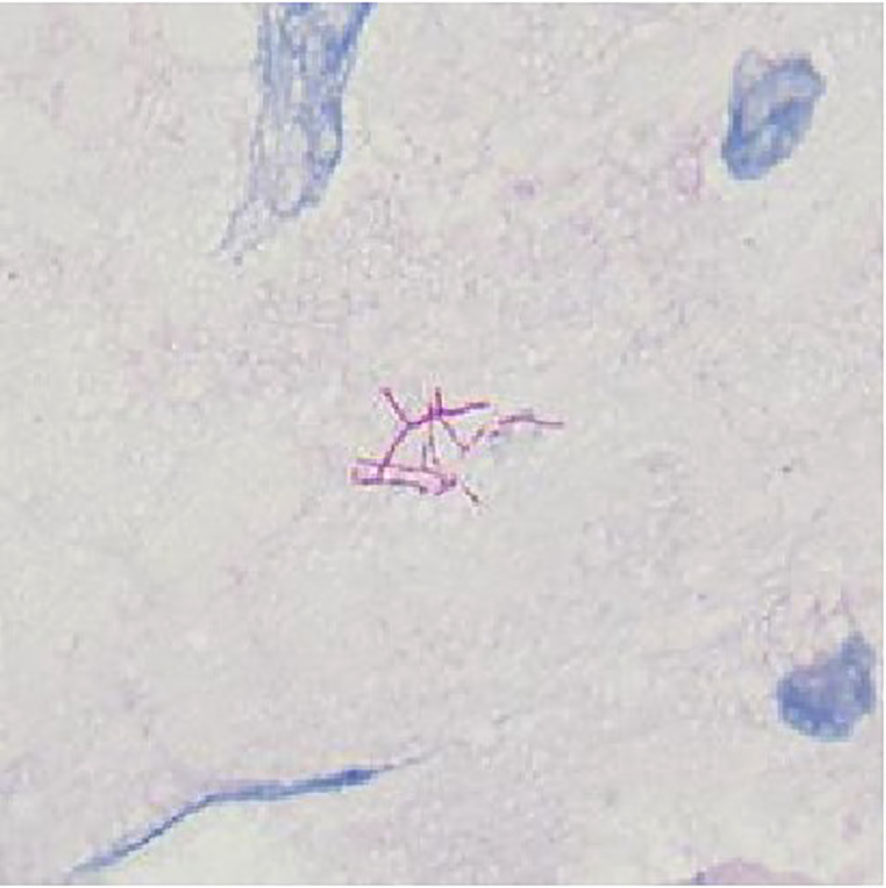
Direct modified acid-fast stain of BALF. Branching filaments were staining modified acid-fast positive.

To identify the infectious pathogens, bedside bronchoscopy was performed on September 19. Bronchoscopic examination revealed mild mucosal congestion and edema. BALF was collected for mNGS and microbial culture. mNGS analysis identified *S. maltophilia* (175,079 reads, 29.1% coverage), *N. cyriacigeorgica* (39,897 reads, 16.6% coverage), and HCMV (6,188 reads). Microbial culture further confirmed the presence of *S. maltophilia* and Nocardia species in the BALF. Antimicrobial susceptibility testing for S. maltophilia demonstrated sensitivity to trimethoprim-sulfamethoxazole (TMP-SMX), levofloxacin, ticarcillin/clavulanate, ceftazidime, and minocycline. Due to the unavailability of appropriate methods, antimicrobial susceptibility testing for Nocardia was not performed. BALF smear microscopy identified modified acid-fast positive Nocardia sp. with characteristic branching and filamentous structures (**Figure 2**). However, no Gram-negative bacilli were observed microscopically, and it is highly probable that the 7-day empirical antimicrobial therapy prior to bronchoscopy significantly reduced the bacterial load of *S. maltophilia*. In summary, the diagnosis was a pulmonary mixed infection involving *N. cyriacigeorgica*, *S. maltophilia*, and HCMV in a patient with MCNS. The treatment regimen was revised to include linezolid (600 mg q12h IV) combined with TMP-SMX (SMX 400 mg + TMP 80 mg, 3 tablets q6h orally). Given the patient’s history of kidney disease and prolonged corticosteroid use, which induced immunosuppression, ganciclovir (300 mg q12h IV) was initiated for HCMV treatment, with a planned discontinuation after two weeks.

After one week of treatment, the patient’s body temperature and inflammatory markers normalized, and symptoms of wheezing, dyspnea, and expectoration were markedly improved. Chest CT performed on September 25 revealed a reduction in the right lung lesion, resolution of ground-glass opacities in both lungs, and a decrease in cavity size (**Figure 1C**). After two weeks of intravenous linezolid and TMP-SMX therapy, both medications were switched to oral administration. Two weeks later, linezolid was discontinued, while TMP-SMX (two tablets orally every six hours) was continued. At the three-month follow-up, chest CT scans demonstrated progressive closure of multiple nodules and cavities (**Figure 1D**). Consequently, the TMP-SMX dosage was adjusted to a prophylactic regimen. Due to the patient’s resumption of corticosteroid therapy for NS, TMP-SMX (1 tablet q12h orally) was prescribed as a prophylactic treatment and continued thereafter. After one year of follow-up, the patient remained in stable condition with no recurrence of infection.

## Discussion

MCNS represents a prevalent etiology of NS. Epidemiological studies indicate that MCNS accounts for 70%-90% of pediatric NS cases (1) and 10.9%-38.5% of adult NS instances (3, 4). The cardinal clinical features of NS encompass massive proteinuria (>3.5 g/24 h) and hypoalbuminemia (<30 g/L), potentially accompanied by edema and hyperlipidemia (5). Histopathological examination reveals essentially normal glomeruli under light microscopy with negative immunofluorescence, while electron microscopy demonstrates diffuse foot process effacement as the pathognomonic morphological feature of MCNS (1). Prednisone or its active metabolite prednisolone, is the cornerstone of early treatment for MCNS. Additionally, frequent relapses often require second-line immunosuppressants to reduce hormone dependence (1). The therapeutic use of corticosteroids and immunosuppressants, while effective, predisposes patients to immunosuppression-related complications, particularly opportunistic infections (2). Notably, PubMed records only one documented case of *Nocardia* infection in MCNS patients (6), with no reported instances of *S. maltophilia* or HCMV co-infections. This report presents the inaugural documented case of pulmonary co-infection involving *N. cyriacigeorgica*, *S. maltophilia*, and HCMV in a patient with MCNS.

*N. cyriacigeorgica* is an aerobic, Gram-positive, modified acid-fast positive, branching bacterium that is ubiquitously distributed in soil and aquatic environments worldwide. This pathogen can cause multi-systemic infections, most commonly affecting the lungs, brain, and skin. Pulmonary involvement constitutes the predominant site of infection, representing 62%-86% of clinical cases (5). Browne et al. reported that pulmonary infections can disseminate to the brain (7). As an opportunistic pathogen, *N. cyriacigeorgica* poses particular risk to NS patients due to compromised renal function, glucocorticoid therapy, and immunosuppressive treatment (5). *S. maltophilia*, a Gram-negative bacillus, exhibits extensive ecological distribution across soil, plant surfaces, human and animal microbiota, and hospital settings (8). Among non-fermenting bacteria, its prevalence ranks after only to *Pseudomonas aeruginosa* and *Acinetobacter baumannii*. This opportunistic pathogen primarily causes respiratory tract infections, establishing its significance as a nosocomial pathogen. HCMV (human herpesvirus 5), a double-stranded DNA virus, maintains a ubiquitous latent and persistent infection profile, with global seroprevalence rates ranging from 60% to 90% (9). While most infections remain asymptomatic or manifest mild symptoms, immunocompromised individuals face substantial risk of severe infections, organ dysfunction, and mortality. Notably, Xue et al. demonstrated that patients with HCMV pneumonia received significantly higher corticosteroid doses and more frequent immunosuppressant administration compared to HCMV-infected individuals without pneumonia (10).

In the management of *N. cyriacigeorgica* infections, TMP-SMX has consistently demonstrated superior efficacy as the first-line therapeutic agent (5). For severe or disseminated infections, a combination therapeutic regimen centered on TMP-SMX is recommended, supplemented by two or three different classes of additional antimicrobials, including third-generation cephalosporins (e.g., ceftriaxone, cefotaxime), carbapenems (e.g., imipenem, meropenem, ertapenem), amikacin, and linezolid (5). In the management of *Stenotrophomonas maltophilia* infections, the preferred initial approach typically involves a combination regimen based on TMP-SMX. It is recommended to select two antimicrobial agents with demonstrated activity (such as TMP-SMX, minocycline/tigecycline, cefiderocol, or levofloxacin) for combined therapy, maintaining this regimen until clinical improvement is achieved. For exceptional clinical scenarios—including rapid clinical deterioration, failure of alternative treatment strategies, or contraindications to other agents—a combination therapy comprising ceftazidime-avibactam and aztreonam may be considered as a therapeutic alternative (11). Regarding HCMV management, clinicians generally adopt either universal prophylaxis or preemptive therapeutic strategies. The initiation of preemptive antiviral therapy should be based on quantitative PCR detection of CMV DNAemia. Systematic research evidence indicates that setting the preemptive therapy threshold at 2–3 log10 IU/mL significantly reduces the incidence of CMV disease (12). Notably, despite the standardization of viral load reporting in international units, the establishment of a universal viral load threshold remains contentious (13). Ganciclovir is the first-line antiviral treatment for HCMV infection. Depending on clinical circumstances, either intravenous ganciclovir or oral valganciclovir may be selected for therapeutic administration, significantly reducing the risk of HCMV-related complications and enhancing clinical outcomes (14, 15).

The patient manifested a triad of clinical symptoms: persistent cough, hemoptysis, and fever with a peak temperature of 39°C. Initial empirical antimicrobial therapy consisting of piperacillin/tazobactam combined with moxifloxacin was administered for seven days. Despite this intervention, the clinical condition deteriorated precipitously, culminating in mild acute respiratory distress syndrome (ARDS). Serial lung CT imaging revealed progressive bilateral pulmonary involvement characterized by multiple patchy high-density shadows and cavitary lesions. The failure of empirical therapy to control disease progression highlighted the critical need for precise pathogen identification to guide targeted antimicrobial intervention. In this context, mNGS has emerged as an indispensable diagnostic tool, particularly for identifying rare, intractable and emerging pathogens. Consequently, BALF samples were collected for both mNGS analysis and conventional microbial culture. Both diagnostic modalities successfully identified *S. maltophilia* and *N. cyriacigeorgica*, while mNGS uniquely detected HCMV. Further clinical evaluation suggested that the patient’s prolonged glucocorticoid therapy for NS had induced a state of immunosuppression, significantly increasing vulnerability to opportunistic infections.

For the treatment of *N. cyriacigeorgica* and *S. maltophilia*, TMP-SMX represents the therapeutic cornerstone. Accordingly, a targeted antimicrobial regimen combining TMP-SMX with linezolid was initiated to optimize therapeutic efficacy and mitigate resistance development. Regarding HCMV management, while immunocompetent individuals with asymptomatic or mild infections typically do not require antiviral therapy, the patient’s two-month history of MCNS coupled with extended glucocorticoid use necessitated antiviral intervention to improve clinical outcomes. Therefore, ganciclovir (0.3 g q12h IV) was incorporated into the treatment regimen. Following two weeks of this comprehensive antimicrobial strategy, the patient demonstrated gradual temperature normalization and marked improvement in respiratory symptoms, including cough and dyspnea. Follow-up imaging studies showed resolution of bilateral diffuse patchy infiltrates and significant reduction in cavitary lesions. Concomitant decreases in inflammatory markers further confirmed the efficacy of the targeted therapeutic approach. After three months of sustained treatment, complete resolution of the pulmonary infection was achieved. Considering the ongoing steroid therapy for nephrotic syndrome, the TMP-SMX dosage was transitioned from therapeutic to prophylactic levels (1 tablet q12h orally) and maintained. At the one-year follow-up evaluation, pulmonary imaging revealed complete resolution of exudative lesions and cavitary changes, with minimal residual fibrotic strands remaining.

As an untargeted high-throughput technology, mNGS allows comprehensive pathogen detection, offering distinct advantages for diagnosing rare, emerging, and polymicrobial infections (16, 17). This case also demonstrates the perfect combination of mNGS and traditional culture technologies to help accurately capture pathogens. However, several limitations exist: First, detected nucleic acids may originate from non-viable microorganisms, as current analytical methods cannot determine microbial viability. This discrepancy may be attributed to the persistence of non-viable *S. maltophilia* DNA fragments post-treatment, as evidenced by negative microscopic findings despite elevated mNGS read counts in the present case. Technically, false-negative results may arise from various factors, including but not limited to NCBI database inaccuracies and/or inefficient bacterial nucleic acid release caused by suboptimal cell wall lysis protocols during sample preparation (18, 19). Furthermore, exogenous contamination and high host DNA ratios (>90%) may compromise detection reliability, necessitating remove DNA contamination from environmental microorganisms or hosts before data analysis (20, 21). Critically, clinical interpretation of microbial single-nucleotide polymorphisms (SNPs) necessitates validation via large-scale multicenter trials. Given the need for further verification of mNGS accuracy through prospective studies and its substantial cost (22), conventional methods (e.g., PCR, serology) remain first-line for definitive pathogen identification. mNGS is optimally employed when: (1) conventional tests contradict strong clinical suspicion, or (2) rare/novel pathogen screening is required (22).

This case illustrates a rare pulmonary co-infection involving *N. cyriacigeorgica*, *S. maltophilia*, and potential HCMV infection, highlighting the critical need for comprehensive diagnostic strategies in immunocompromised patients with poor response to empirical antimicrobial therapy. The integrated application of imaging studies, conventional bacterial culture, and advanced mNGS technology enables accurate pathogen identification and facilitates targeted therapeutic interventions, ultimately enhancing clinical management and patient outcomes.

## Data Availability

The original contributions presented in the study are included in the article/supplementary material. Further inquiries can be directed to the corresponding authors.

## References

[1] Vivarelli M Massella L Ruggiero B Emma F. Minimal change disease. Clin J Am Soc Nephrol. (2017) 12:332–45. doi: 10.2215/CJN.05000516 PMC5293332 27940460

[2] Guo J Li S Xu S Jiang L Gao E Liu Z. Nocardiosis in patients with nephrotic syndrome: a retrospective analysis of 11 cases and a literature review. Int Urol Nephrol. (2020) 52:731–8. doi: 10.1007/s11255-020-02415-z 32124233

[3] Imtiaz S Drohlia MF Nasir K Salman B Ahmad A. Analysis of renal diseases detected in renal biopsies of adult patients: A single-center experience. Saudi J Kidney Dis Transpl. (2017) 28:368–78. doi: 10.4103/1319-2442.202788 28352022

[4] Chang JH Kim DK Kim HW Park SY Yoo TH Kim BS. Changing prevalence of glomerular diseases in Korean adults: a review of 20 years of experience. Nephrol Dial Transplant. (2009) 24:2406–10. doi: 10.1093/ndt/gfp091 19264742

[5] Cheng Y Wang TY Yuan HL Li W Shen JP He ZX. Nocardia infection in nephrotic syndrome patients: three case studies and A systematic literature review. Front Cell Infect Microbiol. (2021) 11:789754. doi: 10.3389/fcimb.2021.789754 35141169 PMC8819730

[6] Garofalo C De Stefano F Gjeloshi K De Gregorio I Masini F Ricozzi C. Very large abscesses of lower limbs by Nocardia farcinica requiring surgical management in patient with minimal change disease under chronic steroid treatment. Nephrol (Carlton). (2021) 26:843–4. doi: 10.1111/nep.v26.10 34060180

[7] Browne WD Lieberson RE Kabbesh MJ. Nocardia cyriacigeorgica brain and lung abscesses in 77-year-old man with diabetes. Cureus. (2021) 13(11):e19373. doi: 10.7759/cureus.19373 34786274 PMC8577824

[8] Brooke JS. Stenotrophomonas maltophilia: an emerging global opportunistic pathogen. Clin Microbiol Rev. (2012) 25:2–41. doi: 10.1128/CMR.00019-11 22232370 PMC3255966

[9] Watanabe M Davidson L Smith P Castellucio PF Jergovic M Uhrlaub JL. Anti-cytomegalovirus antibody levels stratify human immune profiles across the lifespan. Geroscience. (2024) 46:4225–42. doi: 10.1007/s11357-024-01124-0 PMC11336022 38512581

[10] Xue Y Jiang L Wan WG Chen YM Zhang J Zhang ZC. Cytomegalovirus pneumonia in patients with rheumatic diseases after immunosuppressive therapy: A single center study in China. Chin Med J (Engl). (2016) 129:267–73. doi: 10.4103/0366-6999.174490 PMC4799568 26831226

[11] Tamma PD Aitken SL Bonomo RA Mathers AJ van Duin D Clancy CJ. Infectious diseases society of America 2023 guidance on the treatment of antimicrobial resistant gram-negative infections. Clin Infect Dis. (2023). 18:ciad428. doi: 10.1093/cid/ciad428 37463564

[12] Sadowska-Klasa A Leisenring WM Limaye AP Boeckh M. Cytomegalovirus viral load threshold to guide preemptive therapy in hematopoietic cell transplant recipients: correlation with cytomegalovirus disease. J Infect Dis. (2024) 229:1435–9. doi: 10.1093/infdis/jiad386 PMC11095528 37682870

[13] Preiksaitis JK Hayden RT Tong Y Pang XL Fryer JF Heath AB. Are we there yet? Impact of the first international standard for cytomegalovirus DNA on the harmonization of results reported on plasma samples. Clin Infect Dis. (2016) 63:583–9. doi: 10.1093/cid/ciw370 27307504

[14] Ljungman P de la Camara R Robin C Crocchiolo R Einsele H Hill JA. Guidelines for the management of cytomegalovirus infection in patients with haematological Malignancies and after stem cell transplantation from the 2017 European Conference on Infections in Leukaemia (ECIL 7). Lancet Infect Dis. (2019) 19:e260–e72. doi: 10.1016/S1473-3099(19)30107-0 31153807

[15] Razonable RR Humar A. Cytomegalovirus in solid organ transplant recipients-Guidelines of the American Society of Transplantation Infectious Diseases Community of Practice. Clin Transplant. (2019) 33:e13512. doi: 10.1111/ctr.13512 30817026

[16] Jin W Pan J Miao Q Ma Y Zhang Y Huang Y. Diagnostic accuracy of metagenomic next-generation sequencing for active tuberculosis in clinical practice at a tertiary general hospital. Ann Trans Medicine. (2020) 8:1065–. doi: 10.21037/atm-20-2274 PMC7575944 33145284

[17] Ge M Gan M Yan K Xiao F Yang L Wu B. Combining metagenomic sequencing with whole exome sequencing to optimize clinical strategies in neonates with a suspected central nervous system infection. Front Cell Infection Microbiol. (2021) 11. doi: 10.3389/fcimb.2021.671109 PMC8253254 34222042

[18] Blauwkamp TA Thair S Rosen MJ Blair L Lindner MS Vilfan ID. Analytical and clinical validation of a microbial cell-free DNA sequencing test for infectious disease. Nat Microbiology. (2019) 4:663–74. doi: 10.1038/s41564-018-0349-6 30742071

[19] Breitwieser FP Lu J Salzberg SL. A review of methods and databases for metagenomic classification and assembly. Briefings Bioinf. (2019) 20:1125–36. doi: 10.1093/bib/bbx120 PMC6781581 29028872

[20] Weyrich LS Farrer AG Eisenhofer R Arriola LA Young J Selway CA. Laboratory contamination over time during low-biomass sample analysis. Mol Ecol Resources. (2019) 19:982–96. doi: 10.1111/men.2019.19.issue-4 PMC6850301 30887686

[21] Thomas T Gilbert J Meyer F. Metagenomics - a guide from sampling to data analysis. Microbial Inf Experimentation. (2012) 2(1):3. doi: 10.1186/2042-5783-2-3 PMC3351745 22587947

[22] Liu Y Ma Y. Clinical applications of metagenomics next-generation sequencing in infectious diseases. J Zhejiang University-SCIENCE B. (2024) 25:471–84. doi: 10.1631/jzus.B2300029 PMC11199093 38910493

